# Lymphatic imaging to assess rheumatoid flare: mechanistic insights and biomarker potential

**DOI:** 10.1186/s13075-016-1092-0

**Published:** 2016-09-01

**Authors:** Homaira Rahimi, Richard Bell, Echoe M. Bouta, Ronald W. Wood, Lianping Xing, Christopher T. Ritchlin, Edward M. Schwarz

**Affiliations:** 1 Center for Musculoskeletal Research, University of Rochester School of Medicine and Dentistry, Rochester, NY USA; 2 Department of Pediatrics, University of Rochester School of Medicine and Dentistry, Rochester, NY USA; 3 Department of Pathology and Laboratory Medicine, University of Rochester School of Medicine and Dentistry, Rochester, NY USA; 4 Department of Biomedical Engineering, University of Rochester School of Medicine and Dentistry, Rochester, NY USA; 5 Department of Obstetrics and Gynecology, University of Rochester School of Medicine and Dentistry, Rochester, NY USA; 6 Department of Urology, University of Rochester School of Medicine and Dentistry, Rochester, NY USA; 7 Division of Allergy, Immunology, Rheumatology, Department of Medicine, University of Rochester School of Medicine and Dentistry, Rochester, NY USA; 8 Department of Neuroscience, University of Rochester School of Medicine and Dentistry, Rochester, NY USA; 9 University of Rochester Medical Center, 601 Elmwood Avenue, Box 777, Rochester, NY 14642 USA

## Abstract

Proliferation of draining lymphatic vessels coupled with dynamic changes in lymph node volume and flow are characteristic features in rheumatoid arthritis (RA). Furthermore, impaired lymph egress from inflamed synovium is associated with joint flare in murine models of inflammatory-erosive arthritis. Unfortunately, advances towards a greater understanding of lymphatic changes in RA pathogenesis have been slow due to the absence of outcome measures to quantify lymphatic function in vivo. While lymphoscintigraphy is the current standard to assess lymphedema and sentinel lymph nodes in cancer patients, its sensitivity and specificity are inadequate to study lymphatics in RA. The emergence of high-resolution MRI, power Doppler ultrasound, and near-infrared imaging that permits real-time quantification of lymphatic function in animal models has been a major advance, and these techniques have produced a new paradigm of altered lymphatic function that underlies both acute arthritic flare and chronic inflammation. In acute flare, lymphatic drainage increases several fold, whereas no lymphatic contractions are detected in lymph vessels draining chronic arthritic joints. Moreover, these outcomes are now being adapted to study lymphatics in RA towards the development of novel biomarkers of arthritic flare and the discovery of new therapeutic targets. In particular, interventions that directly increase lymphatic egress from diseased joints by opening collateral lymphatic vessels, and that restore lymphatic vessel contractions, provide novel therapeutic approaches with potential for minimal toxicity and immunosuppression. To summarize the origins of this field, recent advances, and future directions, we herein review: current knowledge of lymphatics in RA based on classic literature; new in-vivo imaging modalities that have elucidated how lymphatics modulate acute versus chronic joint inflammation in murine models; and how these preclinical outcome measures are being translated to study lymphatic function in RA inflammation and how effective RA therapies alter lymphatic flow and lymph nodes draining flaring joints.

**Trial registration: **ClinicalTrials.gov NCT02680067. Registered 7 December 2015; ClinicalTrials.gov NCT01098201. Registered 30 March 2010; and ClinicalTrials.gov NCT01083563. Registered 8 March 2010.

**Electronic supplementary material:**

The online version of this article (doi:10.1186/s13075-016-1092-0) contains supplementary material, which is available to authorized users.

## Background

Rheumatoid arthritis (RA) is a chronic, debilitating disease affecting over 1 million people in the United States, and 1 % of the population worldwide [1]. In the current etiological paradigm, autoimmunity to citrullinated peptides drives a systemic inflammatory response that is expressed most strongly in the joints, although a range of other tissues can be affected. However, this model does not explain the events that drive episodic occurrences, known clinically as arthritic or rheumatic flares, and remissions, a hallmark of this disease. RA can also be viewed as a multifactorial syndrome that arises from an interaction of predetermined and stochastic factors which foster disease onset and persistence [2]. This perspective highlights the major unmet clinical burden shouldered by up to 30 % of RA patients refractory to current therapies, and by the larger population that suffers from sudden exacerbation of joint pain which may be accompanied by aggressive cartilage and bone catabolism. Although ubiquitously used in both clinical and preclinical studies [3–5], characterizing the term “flare” has been challenging; however, current efforts are underway to create a uniform set of parameters to define RA flare [6]. What is widely accepted is that flare can occur in newly diagnosed RA patients or in patients with long-standing, chronic arthritis. It is important to note that these enigmatic flares often appear in the setting of long-term effective therapy, sometimes without detectable changes in systemic inflammatory status as measured by inflammatory markers. To better understand whether local factors can trigger arthritic flare, investigators turned their attention to lymphatic drainage, and demonstrated that changes in lymphatic vessel structure and function may be instrumental in provoking joint inflammation [7, 8]. These discoveries were facilitated by novel imaging methods that provided quantitative assessment of lymphatic vessel function for the first time. These advances provide new opportunities to understand how lymphatic biomarkers may be applied to aid in diagnosis of arthritic flare and to assess therapeutic effectiveness. The mechanistic pathways that maintain lymph flow provide new therapeutic targets which may restore dysfunctional lymphatics. In this review, we will: outline the current knowledge of lymphatic function as it relates to RA; describe how contemporary lymphatic imaging methods reveal a central role for lymphatics in arthritic flare; and point out the diagnostic and therapeutic potential of emerging lymphatic vessel biomarkers in RA.

### Lymphatic vasculature structure and function

It is important to understand normal lymphatic vasculature anatomy in order to appreciate when lymphatic dysfunction occurs. A primary purpose of the lymphatic vasculature is to preserve fluid homeostasis within an organism [9]. Lymphatic vessels allow for reabsorption of extravascular and interstitial fluid via blind-ended lymphatic capillaries that become more organized collecting lymphatic vessels. These vessels can drain to lymph nodes (LNs) and are termed afferent vessels. Collecting vessels that drain lymph from a LN are termed efferent vessels. In humans, most of the vessels and LNs in this network ultimately reach the thoracic duct, which collects the lymph and drains it into the blood circulatory system via the left subclavian vein. A small portion of lymph fluid is collected by the right lymphatic duct, which reaches the blood circulation via the right subclavian vein [10]. Most mammals, including mice and rats, have a similar anatomy—that is, thoracic duct drainage of lymph into the blood vasculature [11]—and thus are useful preclinical disease models. Lymph movement results from multiple mechanisms including intrinsic contraction of collecting vessels, as well as the presence of valves in collecting vessels that prevent retrograde movement and thus promote forward movement of lymph. Lymph movement also occurs via an intrinsic contraction associated with movement of lymph as boluses; the mechanisms underlying and regulating this contractile activity remain unclear and are an area of intense study.

### Mechanisms of lymphangiogenesis in inflammation

Research did not focus on the development and growth of lymphatic vessels until the mid-1990s. Initial discoveries by Alitalo and colleagues found vascular endothelial growth factor receptor 3 (VEGFR-3) as a marker for lymphatic vessels in mouse and human tissue, and lent credence to the theory of a venous origin for lymphatic tissue; shortly thereafter, further research from the group identified VEGF-C as the specific ligand for VEGFR-3 [12, 13]. Work from our group further delineated the mechanism of lymphangiogenesis in inflammatory arthritis [14–16]. In the tumor necrosis factor-transgenic (TNF-Tg) mouse, we found increased expression of VEGF-C and increased lymphatic vessel formation in the synovium of these mice early on during the disease process. At 2.5 months old, when clinical parameters such as paw swelling are beginning, the TNF-Tg mice were treated with VEGFR-3 blocking antibody to inhibit VEGF-C binding. After 8 weeks of treatment, we noted decreased lymphatic vessel formation and popliteal lymph node (PLN) size, and increased evidence of joint inflammation as determined by synovial volumes and synovial inflammatory areas at the ankle and knee joints. These findings led us to conclude that VEGF-C-induced lymphangiogenesis ameliorates joint tissue damage in arthritis by promoting drainage of inflamed joints. Furthermore, our in-vitro studies showed that TNF stimulates osteoclast precursors to express VEGF-C and promote lymphangiogenesis. However, persistent inflammation may inhibit lymphatic vessel development and draining function. Recently, we found that TNF stimulates lymphatic endothelial cells to produce iNOS, which inhibits lymphatic smooth muscle cell contraction and lymph drainage [17]. An early study by Polzer et al. [18] reported that specific blockade of TNF was associated with increased lymphangiogenesis in a TNF-Tg mouse model and in RA and spondyloarthritis synovial tissues, suggesting a parallel mechanism in human disease [18]. They theorized that formation of more lymphatic vessels would improve clearance of pathogenic mediators, including proinflammatory cytokines and effector immune cells, such as macrophages. It should be noted that they used a different model of the TNF-Tg mouse, which develops severe inflammatory disease earlier than our model. The finding that both TNF and TNF inhibition can stimulate lymphangiogenesis, although counterintuitive, may therefore relate to the location, stage, and extent of inflammation. It is clear, however, that further research will be necessary to clarify the role of TNF in lymphangiogenesis and inflammation.

### Lymphatics in RA

Although Chauffard and Ramond first described LN involvement and lymphadenopathy in RA patients in 1896 [19, 20], remarkably little is still known about the pathologic features and underling etiology. Harold Paulus and colleagues proposed that effector lymphocytes in lymphatic fluid were central to RA pathogenesis. In support of this view was the dramatic improvement of joint symptoms in RA patients who underwent drainage of the thoracic duct [21]. They described a cohort of nine women with untreated RA aged 24–65 years old who underwent insertion of a fistula into the thoracic duct and drainage of lymphatic fluid. Cells were removed from the fluid and reinfused; the depletion was performed daily for 19–105 days. Within 1 week, the investigators documented improvement in the number of tender and swollen joints, decreased morning stiffness, and improved grip strength. Unfortunately, symptoms returned after removal of the fistula and completion of the study. In subsequent experiments they infused autologous labeled lymphocytes, and arthritic flares were noted a mean of 3.5 days after administration. These findings supported their hypothesis that lymphocytes in the lymphatic fluid are the primary mediators of RA [22–24]. Despite the new knowledge gained in these studies, further advances using this approach were stalled due to the lack of visual and quantitative assessments coupled with the challenges related to the feasibility of the procedure and transient responses. Indeed, the entire literature on lymphatic outcomes in arthritis is limited to three peer-reviewed publications from 1937 to 2001 [25–27].

### Classical lymphoscintigraphy imaging of arthritis in the clinic

Historically, performance of lymphoscintigraphy in arthritis patients was limited to those with lymphedema. In 1968, Kallioma and Vastamak [28] reported on lymphoscintigraphy studies in Finland of the lymphatics in two women with RA who subsequently developed lymphedema of the right arm. They noted decreased uptake in the axillary nodes of the lymphedematous arthritic hand at both timepoints, and proposed an association between lymphatic dysfunction and inflammatory arthritis. Subsequently, radiocolloid lymphoscintigraphy became the imaging modality of choice over the lymphogram, an invasive procedure that requires cannulation and injection of lymphatic vessels with contrast in order to visualize them with X-ray imaging [29].

In the 1990s, several studies used ^99^technetium (^99^Tc) colloid lymphoscintigraphy to demonstrate lymphatic dysfunction in patients with RA or psoriatic arthritis who developed lymphedema in their arthritic extremities [30–33]. In the majority of these case reports, lymphedema lessened when arthritis improved after therapy; this was associated with normalization of colloid uptake assessed by lymphoscintigraphy. However, improvement was not associated with the duration of lymphedema, arthritis, or the type of disease-modifying anti-rheumatic drug (DMARD). The co-occurrence of RA and lymphedema is a relatively rare event so opportunities to gain additional insights are limited; gaining more understanding of the role of lymphatics in RA requires an alternative strategy.

**Fig. 1:**
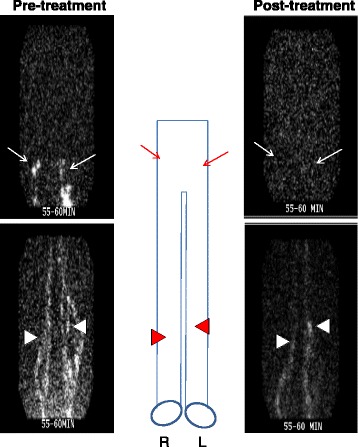
Increased lymphatic flow from flaring joints normalizes with effective anti-TNF therapy. Images of ^99^Tc sulfur colloid tracing of the lower extremity to the pelvis obtained from a RA patient with asymmetric knee flare enrolled in a clinical study to assess the effects of anti-TNF therapy on arthritis and lymphatics (ClinicalTrials.gov NCT01083563). Note the bright ^99^Tc sulfur colloid signal in the inguinal (*arrows*) and popliteal (*arrowheads*) areas on both lower extremities pre treatment, and the decreased signal post treatment, which resulted in normalization of the tt from 15 minutes to 60 minutes. Schematic diagram between the lymposcintigrams orients the regions of interest. *R* right leg, *L* left leg

In our initial human studies we turned to lymphoscintigraphy to analyze lymphatic transport in RA inflammation pre and post anti-TNF therapy as part of a clinical study (ClinicalTrials.gov NCT01083563). The duration of treatment was 18 weeks, and patients were scanned just prior to initiation of therapy and after 18 weeks. An example of these ^99^Tc sulfur colloid tracing studies is shown in Fig. 1. Colloid was injected in the usual fashion into the web spaces of the fingers or toes by a radiologist, and the limb was scanned at specific timepoints. Because lymphoscintigraphy cannot identify individual lymphatic vessels, it cannot detect lymphatic contractions. The technique therefore relies on the transit time (tt) of colloid from the injection site to LN regions, and compares the tt with known normal times. This study produced interesting findings, but we abandoned this approach due to the relatively invasive nature of the technique, radiation exposure, and the prolonged duration of the procedure; these considerations limit its use in the long-term, large-scale studies necessary to further understand the role of lymphatic flow during flare.

### Contrast-enhanced MRI of draining LNs

#### MRI in animal models

To formally address the barriers of real-time in-vivo imaging of lymphatics in RA patients, we turned to contrast-enhanced (CE) MRI to assess the natural history of inflammatory-arthritis in the TNF-Tg and K/BxN murine models of inflammatory-erosive arthritis [34–36]. Although MRI methods to assess synovial inflammation in arthritis were well established [37], visualization of lymphatics was never documented. Subsequent CE-MRI studies in TNF-Tg (3647 line) mice revealed that the draining LN undergoes dynamic transitions which are associated with activity of the arthritis in the proximal joint [38–40]. In the initial “expanding” phase, lymphatic fluid flows at an increased rate and volume from an affected joint to the draining LN via afferent lymphatic vessels. The LN volume expands, presumably to accommodate the influx of cells and lymph exiting the inflamed synovium. Furthermore, a subset of B cells, termed B-cells-in-inflamed-nodes (B-in cells), accumulates in follicular areas of the draining LN [41, 42]. Following an extended expanding phase (typically from 3 to 6 months of age in this model), a stochastic event leads to asymmetric “collapse” of the LN and lymphatic vessels in series along the ipsilateral axis [42, 43]. Coincident with the collapse, B-in cells translocate to the paracortical sinuses of the LN, the proximal joint swells, and joint histopathology shows synovitis. Additionally, intravital immunofluorescent microscopy studies pre and post treatment with anti-CD20 antibodies that ameliorated arthritic inflammation in TNF-Tg mice demonstrated that translocated B-in cells effectively block passive lymph flow through the LN. Further support of the important function of the B-in cells was the restoration of lymphatic flow by B-cell depletion therapy [38]. Interestingly, while B-cell depletion allowed for restoration of passive flow and amelioration of synovial inflammation, it did not restore the lymphatic contractions or active lymphatic transport. Thus, B cells play a passive role in an arthritic flare that lessens in severity when passive lymphatic egress from inflamed joints occurs. This finding suggested two novel intervention strategies currently under investigation: treatment with agents that restore lymphatic contraction and active lymphatic flow; and nonimmunosuppressive treatments that open collateral lymphatic vessels to restore local lymphatic egress by circumventing the sites of B-cell obstruction. A third concept arising from these studies focused on the involvement of lymphatic smooth muscle cells in dysfunction of lymphatics in RA inflammation. Molecular mechanisms responsible for impaired lymphatic smooth muscle cells and their progenitors/stem cells in RA are another active research focus.

#### MRI in clinical studies

**Fig. 2:**
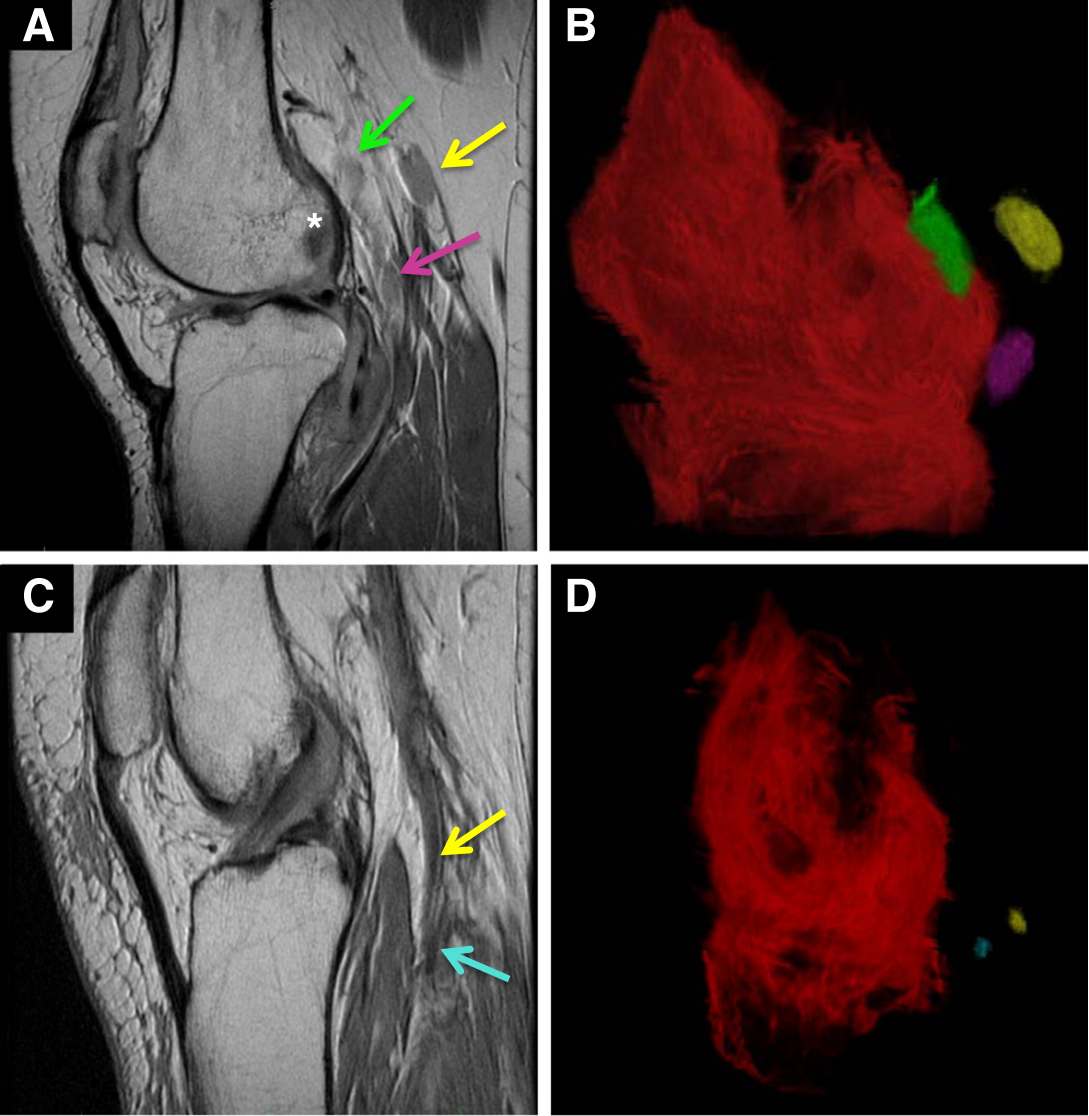
Expanding versus collapsed popliteal LNs in early versus late-stage RA knee flare. MRIs were obtained from RA patients with new-onset (**a,b**) and long-standing (>20 years) (**c,d**) asymmetric knee flare, who were enrolled in a phase 4 clinical trial to assess the effects of anti-TNF therapy on PLN volume (ClinicalTrials.gov NCT01098201). The 2D sagittal views (**a,c**) and 3D volume renderings (**b,d**) illustrate the dramatic differences between early RA flare with expanded PLN (*pink*, *green* and *yellow arrows* corresponding to pseudocolored PLN) adjacent to highly inflamed synovium and focal erosions in the lateral femoral condyle (*), compared with the barely visible collapsed PLN (*blue and yellow arrows* corresponding to pseudocolored PLN) adjacent to advanced pannus tissue and extensive bone erosions

To test whether RA pathogenesis also involves expanding and collapsed draining LNs, we completed a clinical pilot study in which patients with knee flare were imaged with CE-MRI pre and post certolizumab (CZP) therapy (ClinicalTrials.gov NCT01098201). Consistent with the natural history of inflammatory-erosive arthritis in murine models, 2D CE-MRI of patients with newly diagnosed RA who had a flare was associated with highly vascular knee synovitis, bone marrow changes, and very large “expanded” PLN (Fig. 2a). The 3D rendering for volumetric analyses confirmed this finding (Fig. 2b). In contrast, 2D MRI of patients with chronic arthritis (disease duration > 20 years) who developed a flare revealed end-stage synovitis adjacent to focal bone erosions and tiny PLN (Fig. 2c). Volumetric analysis confirmed the predicted “moth-eaten” appearance of the synovium, where vascularity and perfusion is compromised by interdigitating necrotic pannus tissue, and “collapsed” PLN (Fig. 2d). Quantitatively, the total LN volume in 7 of 10 patients with measurable LN on CE-MRI decreased 16 weeks after CZP therapy (mean decrease 37 %; *p* < 0.002). Significant improvement in knee pain measured by the Rheumatoid and Arthritis Outcome Score for the knee [44] inversely correlated with decrease in PLN volume (*R* ^2^ = 0.94). Collectively, these data indicate that RA patients who sustain draining LN volume during anti-TNF therapy experienced the greatest pain relief in flaring joints, and provide the first clinical evidence to support the concept that dynamic changes in LN volume correlate with response to therapy in RA.

#### MRI of lymphatics as biomarkers

Based on the aforementioned preclinical and clinical studies, there is reasonable evidence to support the use of MRI in evaluating draining LNs as a potential early marker of arthritic flare. While the murine models of arthritis have more fully defined that collapse of a draining LN is associated with arthritic flare, a similar correlation in humans has yet to be elucidated. We know, for example, that LNs undergo dramatic changes during synovial inflammation [45], and that these changes can differentiate between RA versus osteoarthritis. However, MRI alone cannot currently evaluate lymphatic vessels, which may be a potential limitation of this imaging modality.

### Power Doppler ultrasound

MRI is the gold standard for quantitative deep soft-tissue imaging due to its unparalleled sensitivity, specificity, and clinical validation [46, 47]. However, the prohibitively high costs of MRI and limited access have contributed to a marked increase in ultrasound (US) imaging. US has several advantages including real-time imaging, accessibility, cost-efficiency, and absence of ionizing radiation [48]. Moreover, US is particularly useful for assessment of RA joint inflammation with gray-scale imaging, and power Doppler (PD) US assessment of synovial hyperemia; both modalities assist in the diagnosis of active synovitis and flares while providing semiquantitative analyses (PD Score) of synovial inflammation for clinical research [49–52]. MRI cost-efficiency issues also apply to preclinical research [53–56], so we adapted PD-US imaging to murine models of inflammatory-erosive arthritis to assess synovitis and alterations of draining LNs longitudinally. Recently, we validated our PD-US quantitative assessments of expanding vs collapsed PLN in TNF-Tg mice [57, 58]. However, PD-US imaging of human LN, particularly PLN, remains elusive due to tissue depth concerns, and continues to be an area of active investigation.

### Near-infrared imaging of indocyanine green

The availability of near-infrared (NIR) imaging of injected indocyanine green (ICG) fluorescent dye was a transformative advance in the quantitative assessment of in-vivo lymphatic function. Low-resolution NIR-ICG imaging has been used to identify sentinel LN during tumor resection surgery for a decade [59, 60], but appropriate optics with computer analyses to quantify human lymphatic function was introduced only recently. In these pioneering studies, groups such as that of Sevick-Muraca and colleagues applied NIR-ICG imaging techniques to evaluate lymphatic vessels in patients with lymphedema [61–63]. More recently, they examined 24 patients with lymphedema and 20 healthy controls, and demonstrated more tortuous and friable lymphatic vessels with extravascular accumulation of lymph in lymphedema patients compared with controls [64]. Furthermore, they quantified the contraction rate of the intrinsic contractile activity of lymphatic vessels in healthy patients. However, they were unable to calculate the contraction rate in lymphedema patients due to the dysmorphic vessel architecture and the inability to visualize ICG-labeled lymph movement in bolus formation, which is how the lymphatic contractions are quantified. Moreover, NIR-ICG imaging actually showed some retrograde movement of lymph in the lymphedema patients. This finding is likely due to the loss of normal vessel contractile ability followed by extravasation of lymph into the surrounding tissue and chronic tissue edema in these patients. A major question we plan to address is whether disturbances in RA lymphatic function are analogous to those observed in lymphedema patients.

The first publication describing NIR-ICG imaging in arthritis used infrared spectroscopy to study synovial fluid in patients with inflammatory arthritis versus osteoarthritis in order to differentiate the types of arthritis [65]. Preclinical studies using mouse models of arthritis reported that in-vivo arthritis detection in the early stages of disease activity is feasible with the use of NIR imaging and fluorescent-labeled molecules such as ICG and folate receptor-targeted dyes [66–68]. Moving towards clinical translation, Krohn et al. [69] examined the hands of 31 patients with untreated early RA using NIR-ICG optical imaging versus conventional MRI and US. Patients were given intravenous ICG and were assessed with a specialized imaging system and camera. They examined wrist, metacarpophalangeal (MCP), and proximal interphalangeal (PIP) joints and developed a 4-point scoring system to identify the degree of enhancement. The findings were correlated with MRI scoring of synovitis in the same joints. The results showed that NIR-ICG imaging correlates well with MRI findings at certain phases of ICG uptake, but it was inconsistent and not superior to current imaging modalities. However, the relative speed and simplicity of this minimally invasive procedure coupled with in-vivo real-time results demonstrated the potential of providing information to the clinician in a timely manner.

**Fig. 3:**
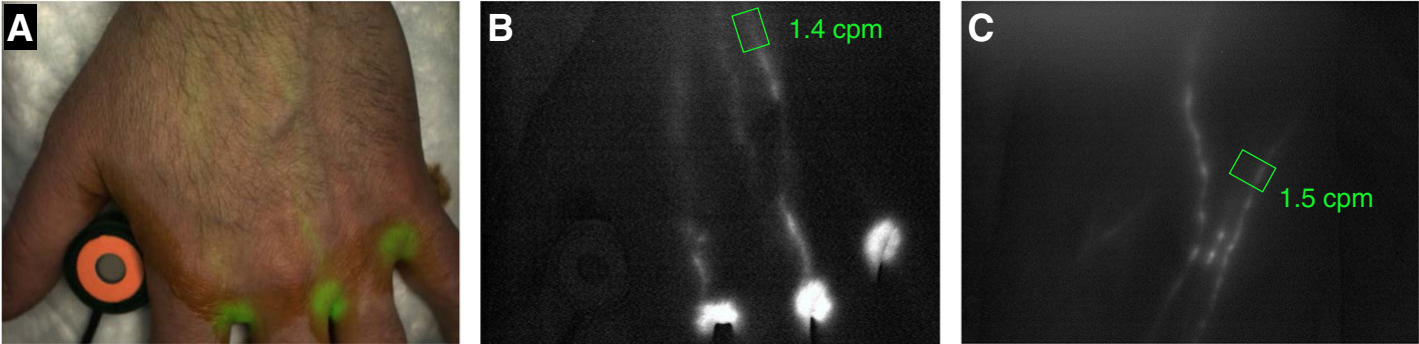
Clinical NIR imaging to quantify lymphatic flow in the upper extremity. A custom NIR imaging system (FD-1665; FluxData Inc., Rochester, NY, USA) was used to assess lymphatic contraction frequency in a healthy human subject after ICG injection in the second, third, and fourth web spaces of both hands as described in ClinicalTrials.gov NCT2680067. NIR excitation (<1.8 mW/cm^2^) was monitored with a Thorlabs PM16-121 power meter adjacent to the first web space. After injections, the upper extremities were imaged for 10 minutes to observe lymphatic flow. Visible and NIR (>800 nm) images were collected simultaneously; ICG fluorescence images were used to pseudocolor the visible image to provide anatomic localization (**a**). The ROI was positioned over lymphatics to calculate the mean pixel value for each NIR frame; peaks associated with lymphatic contractions were counted to calculate cpm. Representative images of the left hand (**b**) obtained from real-time video (Additional file 1: Movie 1) and right antecubital fossa (**c**) (Additional file 2: Movie 2) with the ROI and the respective cpm (*green*)

We are now performing a clinical pilot study of NIR-ICG imaging of the upper extremities in RA patients experiencing hand and/or wrist joint flares, and comparing the lymph flow data with that observed in healthy controls, to evaluate lymphatic vasculature and LNs in human subjects (ClinicalTrials.gov NCT02680067). In brief, a custom NIR imaging system is used to visualize lymphatic contractions after a negligible amount of ICG dye is injected into the web spaces of the hands. NIR excitation is monitored using a power meter. After the injections, the upper extremities are imaged continuously for 10 minutes to observe lymphatic flow. A region of interest (ROI) is then positioned over lymphatics to calculate the mean pixel value; peaks associated with lymphatic contractions are indicative of contractions per minute (cpm). Figure 3 and Additional file 1: Movie 1 and Additional file 2: Movie 2 describe the technique and illustrate the sensitivity and specificity of the approach to quantify lymphatic contractions using this first-generation experimental approach.

The rationale for using NIR-ICG to evaluate lymphatic contractions is reasonable because it provides real-time information for the clinician. Furthermore, dysfunctional contractions, or no contractions, could indicate that the arthritic episode is the result of a drainage issue rather than synovial disturbances. The treatment would thus be tailored appropriately (i.e. with use of anti-TNF therapy), which has been associated with increased lymphatic angiogenesis [18] rather than DMARDs or steroids. Because of the extensive use of ICG in cardiovascular imaging over the decades, this dye has been studied extensively in humans and has been approved by the FDA for use as an imaging dye. There is therefore no major health limitation of the NIR-ICG technique. The only limitation is the duration of a study, which can take about 1–2 hours, the equivalent of current imaging techniques such as MRI.

## Conclusions

The potential importance of lymphatic function as a key variable in RA flare is supported by the presence of palpable LN and lymphedema in some patients, and by preclinical data demonstrating major alterations in draining LNs and vasculature prior to arthritis onset. The role of the lymphatics in RA can now be examined with the advent of in-vivo imaging modalities that quantify lymphatic flow and contraction frequency. These technical advances may empower investigators to promote understanding in three critical areas. The first area is the redefinition of patterns of lymphatic flow anatomically, because most of our knowledge is derived from cadaveric studies and circulatory system mapping. Interestingly, our early studies have demonstrated inconsistencies in lymphatics drainage compared with previously published data. The second area is discovery of the cellular, molecular, and structural mechanisms that regulate lymphatic function and that are closely integrated with local biomechanics, inflammation, and parasympathetic innervation. Finally, the ultimate goal of this research is to identify novel molecular targets that will give rise to new interventions for RA flare. To achieve this goal, advancing technologies to noninvasively evaluate superficial and deep lymphatics in humans is a critical first step.

## Additional files

Additional file 1: Video 1 of the left hand of a healthy patient after injection of ICG into the web spaces. The synchronized chart below is the mean fluorescent intensity (arbitrary units) within the *green* ROI. 20× real time. (M4V 2.64 mb)
Additional file 2: Video 2 of the right arm of a healthy patient after injection of ICG into the web spaces. The synchronized chart below is the mean fluorescent intensity (arbitrary units) within the *green* ROI. 20× real time. (M4V 2.18 mb)

## Abbreviations

^99^Tc: Technetium99
B-in cells: B-cells-in-inflamed-nodes
CE: Contrast enhanced
CZP: Certolizumab
DMARD: Disease-modifying anti-rheumatic drug
LN: Lymph node
MCP: Metacarpophalangeal
NIR: Near-infrared imaging
ICG: Indocyanine green
PD: Power Doppler
US: Ultrasound
PIP: Proximal interphalangeal
RA: Rheumatoid arthritis
TNF-Tg: Tumor necrosis factor transgenic
tt: Transit time
